# Prognosis in triple‐negative apocrine carcinomas of the breast: A population‐based study

**DOI:** 10.1002/cam4.2634

**Published:** 2019-10-23

**Authors:** Wenyu Wu, Meiying Wu, Guowen Peng, Degang Shi, Jian Zhang

**Affiliations:** ^1^ Department of Oncology Zhujiang Hospital Southern Medical University Guangzhou China; ^2^ Target and Interventional Therapy Department of Oncology First People's Hospital of Foshan Foshan China; ^3^ Department of Cardiothoracic Surgery The Ninth Affiliated Hospital of Guangxi Medical University Beihai China

**Keywords:** apocrine carcinoma, prognosis, SEER database, triple‐negative breast cancer

## Abstract

**Background:**

Triple‐negative apocrine carcinoma (TNAC) of the breast is a very rare type of breast cancer. Furthermore, the clinicopathological features, prognosis, and potential impact of treatment strategies in TNAC remain unclear.

**Methods:**

Data from the Surveillance, Epidemiology, and End Results (SEER) program were used to identify breast cancer patients diagnosed between 2010 and 2016 with TNAC and triple‐negative breast cancer (TNBC, IDC [invasive ductal carcinoma], NOS [not otherwise specified]). Chi‐squared tests were used to examine the categorical variables between the two groups. Overall survival (OS) of TNAC and TNBC was assessed by Kaplan‐Meier analyses and Cox regression. Breast cancer‐specific survival (BCSS) was evaluated by Nelson‐Aalen analyses and competing risk regression.

**Results:**

We identified 31 362 patients from the SEER database, including 366 patients with TNAC and 30 996 patients with TNBC. TNAC was correlated with older age, lower T stage and lower tumor grade. Patients with TNAC had better OS compared with TNBC patients; the 5‐year OS rates were 82.2% vs 73.5% (*P* < .001). The breast cancer‐related death rate was significantly lower in patients with TNAC than in patients with TNBC, with a 5‐year cumulative incidence of 9.1% vs 22.9% (*P* < .001). Chemotherapy was significantly associated with improved OS in TNAC patients, but radiotherapy was not associated with OS in TNAC patients. In the multivariable Cox regression, TNAC was still associated with improved OS (HR [hazard ratio], 0.61; 95% CI [confidence interval] 0.45‐0.83; *P* = .002). In the multivariable competing risk regression, the significantly higher BCSS in patients with TNAC compared patients with TNBC remained (subdistribution HR [SHR], 0.42; 95% CI, 0.27‐0.64; *P* < .001).

**Conclusion:**

Patients with TNAC had a better prognosis than patients with TNBC, and chemotherapy was associated with survival advantages in TNAC patients.

## Graphical abstract

Invasive triple‐negative apocrine carcinoma (TNAC) of the breast is a rare type of triple‐negative breast cancer. Furthermore, the clinicopathological features, prognosis of TNAC, and the potential effect of systemic treatments on TNAC remain unclear. The purpose of the present study was to gain better knowledge of clinicopathological features and survival differences in patients with TNAC and TNBC (non‐apocrine breast cancer), by utilizing a population wide database to enroll a large population of breast cancer.

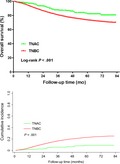

## 1 INTRODUCTION

Invasive apocrine carcinoma (AC), a pathological type of invasive ductal carcinoma (IDC) of the breast, is defined as a breast tumor composed of epithelium with apocrine differentiation in more than 90% of the tumor cells and accounts for 0.3%‐4% of all breast cancer.^1^, ^2^ It is well known that AC tends to represent a unique hormone receptor profile—progesterone receptor (PR)‐negative, estrogen receptor (ER)‐negative, and androgen receptor (AR)‐positive.^3^ The overexpression of human epidermal growth factor receptor 2 (HER2) is common in AC (~30%),^4^, ^5^ but HER2‐negative AC can be phenotyped as triple‐negative breast cancer (TNBC). However, from the management perspective, most AC can be treated as TNBC, thus they are not subjected to standard anti‐HER2 or endocrine treatments. Considering the clinicopathogical features and prognosis, it is reasonable that triple‐negative apocrine carcinoma (TNAC) should be distinguished from TNBC.^6^, ^7^

Because TNAC is a rare pathological type of TNBC, the clinicopathological features and prognosis of these patients have only been reported in a limited number of studies—case reports or studies recruiting a small number of patients. As a result, the prognostic values of clinicopathological features and treatments in TNAC patients remain unclear. An observational study of 46 breast cancer patients showed that AC was more often present in older women with lower grade and T stage compared with TNBC, but some AC patients in this study were non‐TNBC.^8^ Meattini et al showed that TNAC had a favorable overall survival (OS) outcome when compared with other TNBC tumors.^9^ However, this study provided limited information on the prognosis for TNAC due to its small sample size. Two other studies showed that there was no difference in survival between AC patients and non‐AC patients.^10^, ^11^ Consequently, it is important to clarify the clinicopathological features and prognosis of TNAC in a large population.

The purpose of the present study was to investigate the clinicopathological features and survival differences in patients with TNAC and TNBC (IDC, NOS [not otherwise specified]) by utilizing a population‐wide database to enroll a large population of breast cancer patients. OS and breast cancer‐specific survival (BCSS) were compared between the two groups using comprehensive statistical methods with a multivariable Cox model and competing risk regression to adjust for confounding factors. We sought to identify the prognostic factors that might explain the differences in survival between patients with TNAC and TNBC.

## 2 MATERIALS AND METHODS

### 2.1 Study population

Patient data were obtained from the Surveillance, Epidemiology, and End Results (SEER) website (http://seer.cancer.gov/) using SEER*stat version 8.3.5. We used SEER data released in March 2019 and extracted data from 2010 to 2016. As the SEER database began to include HER2 status in 2010, we chose 1 January 2010 as the starting point for the study.

The inclusion criteria were as follows: female, over 18 years of age, unilateral breast cancer, pathologic confirmation of AC (ICD‐0‐3 8401) and IDC, NOS (ICD‐0‐3 8500), triple‐negative breast cancer subtype, breast cancer as first and the only diagnosis, diagnosis not obtained from a death certification or autopsy, known survival time and surgery status, and known T and N stage (with T0 and Tis tumors excluded). Finally, 31 362 patients were included; 366 patients were diagnosed with TNAC and 30 996 with TNBC (Table 1).

**Table 1:** Stepwise inclusion and exclusion counts

Removal criterion	TNAC		TNBC	
	Removed	Remaining	Removed	Remaining
2010‐2016 TNAC or TNBC patients	0	454	0	38 061
Exclude men	0	454	40	38 021
Exclude patients whose tumor was not the first tumor	81	373	6026	31 995
Exclude patients without histology or cytology confirmation	0	373	7	31 988
Exclude patients without survival information/ diagnosed by autopsy/death record only	0	373	0	31 988
Exclude patients younger than 18 y	0	373	1	31 987
Exclude patients whose disease is stage T0/Tis	2	371	45	31 942
Exclude patients with bilateral involvement	0	371	8	31 934
Exclude patients with unknown T and/or N stage	5	366	702	31 051
Exclude patients with unknown surgery status	0	366	55	30 996
Final data set	0	366	0	30 996

The demographic features included age at diagnosis, race, and marital status; the clinicopathological features included tumor grade, breast subtype, laterality, T stage, N stage, metastasis, and American Joint Committee on Cancer (AJCC) stage; and the treatment information included radiation therapy, chemotherapy and surgery. The primary endpoint of the study was BCSS from the date of diagnosis to the date of death from breast cancer, and the secondary outcome was OS from the date of diagnosis to the date of death from any cause. Patients alive at the time of last follow‐up and/or at the end of the analysis period (November 31, 2016) were right censored.

We obtained permission to use data files from the SEER database. Therefore, our study was exempted by the Ethics Committee of Zhujiang Hospital of Southern Medical University.

### 2.2 Statistical analysis

The clinicopathological features were compared between TNAC and TNBC using the chi‐squared test. Categorical variables were reported as the number of cases and percentages. OS rates were calculated by the Kaplan‐Meier analyses, and survival experiences were compared by using the log‐rank test. Multivariable Cox proportional hazard regression was used to evaluate the prognostic factors for OS, and the results were presented with a hazard ratio (HR) and 95% confidential interval (CI).

For the competing risk regression model, the outcome of interest was defined as breast cancer‐specific death, while death not related to breast cancer was considered a competing risk. The cumulative incidence function for breast cancer‐specific death was performed, considering death not related to breast cancer as a competing risk of death. Nelson‐Aalen cumulative risk curves of the incidence function for breast cancer‐specific death were conducted and compared by Gray's test. Fine and Gray's competing risk regression was used to assess the prognostic factors associated with BCSS, with results presented as a subdistribution HR (SHR) and 95% CI.

All statistical analyses were performed using Stata 15.0 (Stata Corporation, College Station, Texas) and R statistical software 3.5.0 (StataCorp LLC, College Station, Texas). All statistical tests were two‐sided, and the level of significance was set at *P* < .05.

## 3 RESULTS

### 3.1 Patient characteristics of TNAC and TNBC

After applying the inclusion and exclusion criteria, the study cohort included 366 patients diagnosed with TNAC and 30 996 patients diagnosed with TNBC (IDC, NOS) from 2010 to 2016 (Table 1).

Patient demographics, clinicopathological features, and treatment information are shown in Table 2 for TNAC and TNBC. There were no significant differences found in marital status, laterality, N stage, metastasis, and radiation therapy when comparing patients with TNAC and TNBC. However, TNAC patients had an older age at diagnosis (≥50 years, 91.0% vs 70.7%, *P* < .001) and had a significantly lower black race prevalence (13.9% vs 21.0%, *P* = .001) than TNBC patients.

**Table 2:** Patient and tumor characteristics in the triple‐negative AC (TNAC) and IDC (TNBC) groups

Characteristic	TNAC (N = 366)		TNBC (N = 30 996)		*P*‐value
	n	%	n	%	
Age (years)					<.001
18‐49	33	9.0	9072	29.3	
≥50	333	91.0	21 924	70.7	
Race					.001
White	265	72.4	21 881	70.6	
Black	51	13.9	6521	21.0	
Other	48	13.1	2406	7.8	
Unknown	2	0.5	188	0.6	
Marital status					.500
Married	185	50.5	16 622	53.6	
Unmarried	161	44.0	12 757	41.2	
Unknown	20	5.5	1617	5.2	
Laterality					.167
Left	170	46.4	15 925	51.4	
Right	196	53.6	15 067	48.6	
Unknown	0	0.0	4	0.0	
Tumor grade					<.001
I/II	242	66.1	5193	16.7	
III/IV	113	30.9	24 878	80.3	
Unknown	11	3.0	925	3.0	
T stage					<.001
T1	224	61.2	13 265	42.8	
T2	108	29.5	13 139	42.4	
T3	23	6.3	2657	8.6	
T4	11	3.0	1935	6.2	
N stage					.296
N0	250	68.3	19 755	63.7	
N1	83	22.7	7816	25.2	
N2	20	5.5	1919	6.2	
N3	13	3.6	1506	4.9	
Metastasis					.070
M0	355	97.0	29 417	94.9	
M1	11	3.0	1579	5.1	
AJCC stage					<.001
I	181	49.5	11 052	35.7	
II/III	174	47.5	18 365	59.2	
IV	11	3.0	1579	5.1	
Radiation therapy					.090
None/unknown	168	45.9	15 609	50.4	
Done	198	54.1	15 387	49.6	
Chemotherapy					<.001
None/unknown	134	36.6	7247	23.4	
Done	232	53.4	23 729	76.6	
Surgery					<.001
None	10	2.7	2487	8.0	
Done	356	97.3	28 509	92.0	

Abbreviations: AJCC, American Joint Committee on Cancer; TNAC, triple‐negative apocrine carcinoma; TNBC, triple‐negative breast cancer.

A higher rate of grade I/II tumors (66.1% vs 16.7%, *P* < .001) was observed in TNAC patients than in TNBC patients. Moreover, TNAC patients presented with a greater frequency of T1 stage (61.2% vs 42.8%, *P* < .001). Consequently, a higher proportion of TNAC patients had AJCC stage I disease compared with TNBC patients (49.5% vs 35.7%, *P* < .001). Treatments were also significantly different between TNAC and TNBC patients. The surgery rate was higher in patients with TNAC compared to patients with TNBC (97.3% vs 92.0%, *P* < .001). In addition, chemotherapy was used less frequently in patients with TNAC than in patients with TNBC (59.8% vs 73.6%, *P* < .001).

### 3.2 Survival analysis

In the current study, the median follow‐up time was 33 months for the TNAC group and 30 months for the TNBC group. There were 1423 (4.6%) breast cancer‐related deaths observed in the TNBC group and 23 (6.3%) in the TNAC group. Deaths from other causes were identified in 4305 (13.9%) patients and 19 (5.2%) patients in the TNBC and TNAC groups, respectively.

**Figure 1:**
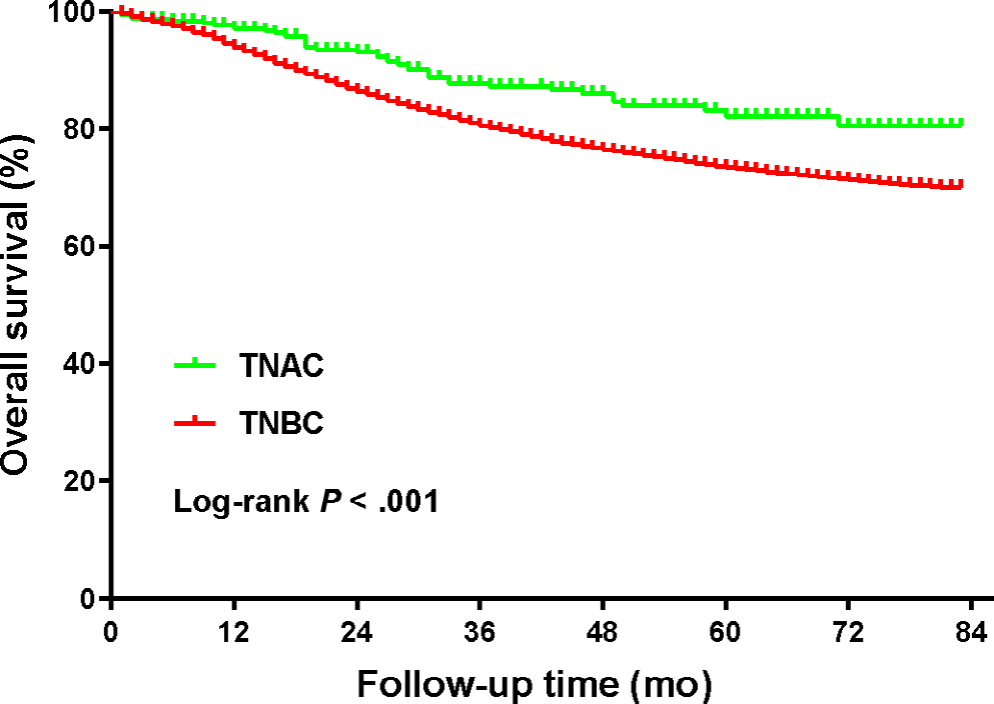
Comparison of overall survival between patients with TNAC and patients with TNBC (31 362 patients in total)

**Figure 2:**
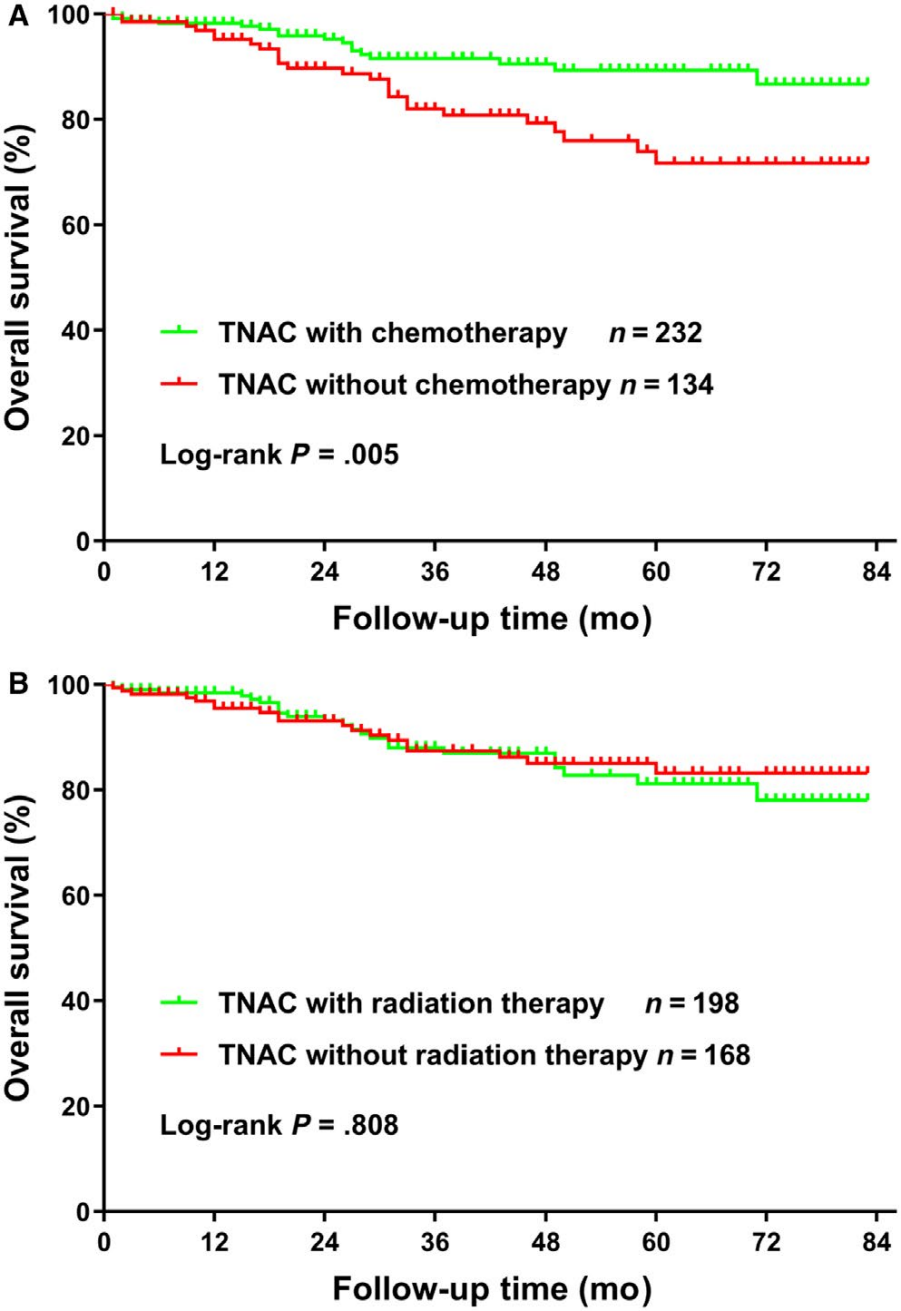
Comparison of overall survival for 366 TNAC patients with or without chemotherapy (A) or radiation therapy (B)

To explore whether patients with TNAC and TNBC had different OS rates, we first compared the Kaplan‐Meier curves and the 5‐year OS rates of patients with TNAC and TNBC. The 5‐year OS rate for all patients was 73.6% (95% CI, 72.9%‐74.2%), with a 5‐year OS rate of 82.2% (95% CI, 76.1%‐86.8%) for patients with TNAC and 73.5% (95% CI, 72.8%‐74.1%) for patients with TNBC. The Kaplan‐Meier curves and log‐rank test showed that TNAC patients had better OS rates compared with TNBC patients (Figure 1, *P* < .001). To further investigate the impacts of chemotherapy and radiation therapy on OS in TNAC patients, a Kaplan‐Meier analysis was applied to the calculated OS rates. From Figure 2A, chemotherapy was significantly associated with improved TNAC OS (*P* = .005), with a 5‐year OS rate of 89.3% (95% CI, 8.27%‐93.5%) for the chemotherapy group and 71.7% (95% CI, 59.9%‐80.5%) for the non‐chemotherapy group. However, radiation therapy was not associated with OS in TNAC patients (*P* = .808, Figure 2B).

**Figure 3:**
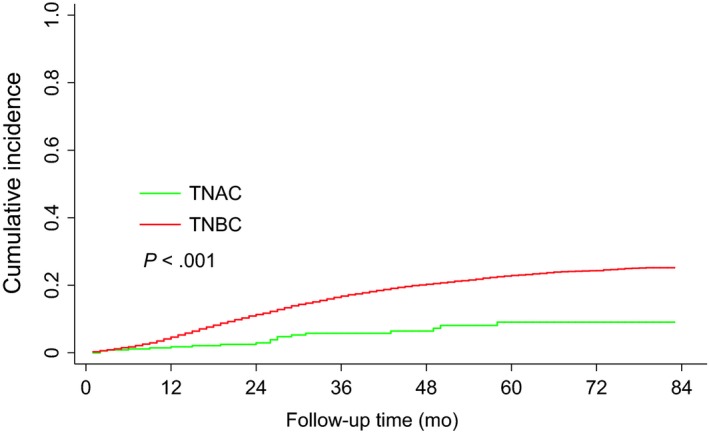
Comparison of the cumulative probability of breast cancer‐specific death between patients with TNAC and patients with TNBC (31 362 patients in total)

Considering deaths unrelated to breast cancer, the cumulative incidence of breast cancer‐related death in all patients over 5 years was 22.7% (95% CI, 22.0%‐23.5%), with a 5‐year cumulative incidence of 9.1% (95% CI, 5.6%‐14.8%) for TNAC and 22.9% (95% CI, 22.2%‐23.7%) for TNBC. As shown in Figure 3, TNAC patients had a lower cumulative incidence of breast cancer‐related death than TNBC patients (*P* < .001).

To adjust for potential confounding factors, including age, race, marital status, tumor grade, T stage, N stage, metastasis, radiation therapy, chemotherapy and surgery, a multivariable Cox regression model was performed. Consistent with the results of the univariable analysis (Table 3), TNAC patients had better OS rates compared with TNBC patients (HR, 0.61; 95% CI, 0.45‐0.83; *P* = .002). In the Cox regression model, older age (*P* < .001), unmarried status (*P* < .001), high‐grade tumor (*P* < .001), advanced T stage (*P* < .001), advanced N stage (*P* < .001), and metastasis (*P* < .001) were independent factors associated with worse OS. However, other races (*P* < .001), radiation therapy (*P* < .001), chemotherapy (*P* < .001), and surgery (*P* < .001) were independent protective factors for OS.

**Table 3:** Univariable and multivariable analysis of overall survival (OS)

Characteristic	Univariable analysis		Multivariable analysis	
	HR (95% CI)	*P*‐value*	HR (95% CI)	*P*‐value*
Age ≥ 50 y	1.26 (1.18‐1.33)	<.001	1.28 (1.20‐1.36)	<.001
Race				
White	1 (reference)		1 (reference)	
Black	1.25 (1.17‐1.33)	<.001	1.03 (0.97‐1.10)	.311
Other	0.79 (0.71‐0.88)	<.001	0.75 (0.67‐0.84)	<.001
Unknown	0.25 (0.12‐0.53)	<.001	0.22 (0.11‐0.47)	<.001
Marital status				
Married	1 (reference)		1 (reference)	
Unmarried	1.66 (1.57‐1.79)	<.001	1.28 (1.21‐1.35)	<.001
Unknown	1.33 (1.19‐1.50)	<.001	1.16(1.04‐1.31)	.012
Tumor grade				
I/II	1 (reference)		1 (reference)	
III/IV	1.27 (1.18‐1.36)	<.001	1.15 (1.07‐1.24)	<.001
Unknown	1.77 (1.53‐2.05)	<.001	1.06 (0.87‐1.17)	.937
T stage				
T1	1 (reference)		1 (reference)	
T2	2.24 (2.09‐2.40)	<.001	1.93 (1.79‐2.07)	<.001
T3	4.92 (4.52‐5.36)	<.001	2.92 (2.65‐3.20)	<.001
T4	10.66 (9.82‐11.57)	<.001	3.67 (3.33‐4.05)	<.001
N stage				
N0	1 (reference)		1 (reference)	
N1	2.70 (2.54‐2.87)	<.001	1.99 (1.86‐2.12)	<.001
N2	4.22 (3.88‐4.59)	<.001	2.92 (2.67‐3.19)	<.001
N3	7.41 (6.83‐8.03)	<.001	3.28 (2.99‐3.61)	<.001
Metastasis	12.02 (11.25‐12.84)	<.001	3.74 (3.45‐4.06)	<.001
AC Histology	0.58 (0.43‐0.78)	<.001	0.61 (0.45‐0.83)	.002
Radiation therapy	0.63 (0.60‐0.66)	<.001	0.80 (0.76‐0.85)	<.001
Chemotherapy	0.65 (0.62‐0.69)	<.001	0.45 (0.42‐0.47)	<.001
Surgery	0.16 (0.15‐0.17)	<.001	0.44 (0.41‐0.48)	<.001

Abbreviations: AC, apocrine carcinoma; CI, confidential interval; HR, hazard ratios; SHR, subdistribution hazard ratio.

* *P* values from the Cox proportional hazard regression model.

Furthermore, considering deaths unrelated to breast cancer, a multivariable Gray's competing risk regression model was performed to adjust for potential confounding factors. Consistent with the results of the univariable analysis, TNAC patients still had better BCSS rates compared with TNBC patients (SHR, 0.42; 95% CI, 0.27‐0.64; *P* < .001) (Table 4). As shown in Table 4, unmarried status (*P* < .001), high‐grade tumor (*P* < .001), advanced T stage (*P* < .001), advanced N stage (*P* < .001), and metastasis (*P* < .001) were significant risk factors for BCSS. In contrast, other races (*P* < .001), radiation therapy (*P* < .001), chemotherapy (*P* < .001), and surgery (*P* < .001) were associated with improved BCSS.

**Table 4:** Univariable and multivariable analysis of breast cancer‐specific survival (BCSS)

Characteristic	Univariable analysis		Multivariable analysis	
	HR (95% CI)	*P*‐value*	HR (95% CI)	*P*‐value*
Age ≥ 50 y	0.97 (0.91‐1.03)	.332	1.07 (0.99‐1.15)	.068
Race				
White	1 (reference)		1 (reference)	
Black	1.28 (1.19‐1.37)	<.001	1.02 (0.94‐1.11)	.667
Other	0.83 (0.73‐0.94)	.004	0.78 (0.68‐0.89)	<.001
Unknown	0.15 (0.05‐0.47)	.001	0.11 (0.03‐0.38)	<.001
Marital status				
Married	1 (reference)		1 (reference)	
Unmarried	1.45 (1.36‐1.54)	<.001	1.12 (1.04‐1.20)	<.001
Unknown	1.16 (1.01‐1.33)	.042	1.09 (0.95‐1.26)	.23
Tumor grade				
I/II	1 (reference)		1 (reference)	
III/IV	1.46 (1.34‐1.60)	<.001	1.19 (1.08‐1.32)	<.001
Unknown	2.07 (1.75‐2.46)	<.001	1.02 (0.82‐1.27)	.864
T stage				
T1	1 (reference)		1 (reference)	
T2	2.78 (2.55‐3.02)	<.001	2.06 (1.88‐2.26)	<.001
T3	6.78 (6.12‐7.49)	<.001	3.27(2.91‐3.67)	<.001
T4	13.57 (12.27‐15.01)	<.001	3.76 (3.30‐4.29)	<.001
N stage				
N0	1 (reference)		1 (reference)	
N1	3.50 (3.26‐3.77)	<.001	2.28 (2.10‐2.48)	<.001
N2	5.62 (5.11‐6.18)	<.001	3.45 (3.09‐3.87)	<.001
N3	10.22 (9.31‐11.22)	<.001	4.01 (3.53‐4. 55)	<.001
Metastasis	12.42 (11.48‐13.45)	<.001	3.61 (3.24‐4.01)	<.001
AC Histology	0.35 (0.23‐0.55)	<.001	0.42 (0.27‐0.64)	<.001
Radiation therapy	0.72 (0.68‐0.76)	<.001	0.85 (0.79‐0.92)	.001
Chemotherapy	1.06 (0.98‐1.13)	.138	0.69 (0.63‐0.76)	<.001
Surgery	0.17 (0.15‐0.18)	<.001	0.48 (0.43‐0.53)	<.001

Abbreviations: AC, apocrine carcinoma; CI, confidential interval; HR, hazard ratios; SHR, subdistribution hazard ratio.

* *P* values from the competing risk regression model.

## 4 DISCUSSION

The diagnosis of AC of the breast has been controversial because of the lack of strict diagnostic criteria.^2^ In the current study, we identified AC patients coded by ICD‐O‐3 8401/3, and the diagnosis is morphologically exactly AC, which is not a carcinoma with apocrine features, differentiation, or type. TNAC is an extremely rare type of triple‐negative breast cancer,^1^, ^9^ so it is quite difficult to obtain a large enough number of these patients in clinical practice. Based on a large population from the SEER database, a retrospective study was performed to explore the clinicopathological features and prognostic factors of TNAC patients. Our study demonstrated that patients with TNAC had better OS when compared with TNBC patients in a multivariable Cox regression analysis. After taking deaths not related to breast cancer into consideration, TNAC patients had significant BCSS benefits compared with TNBC patients. Until now, because there is a shortage of precise prognostic data, TNAC is often grouped with other TNBCs, which usually rely on broad‐spectrum and highly efficient multidrug chemotherapeutic regimens.^12^, ^13^ However, based on the current study, there is reason to believe that treating TNAC like other TNBCs is inappropriate. For example, the most significant distinguishing feature of TNAC is its preference for older women (*P* < .001), a population less likely to tolerate aggressive multidrug chemotherapies.

Similar to our study, a retrospective study of the SEER database from 2003 to 2013 found that OS and BCSS were both worse in AC patients than in patients with IDC in univariable analysis, but it was not found to be an independent prognostic factor in multivariable analysis.^14^ Furthermore, this study investigated the prognosis of molecular subtypes and showed that patients with TNAC presented better OS and BCSS than patients with TNBC in a univariable analysis. However, this study could not analyze TNAC well as the SEER database did not start recording HER2 status until 2010. The recent SEER data including HER2 status provide a unique opportunity to explore the prognosis of patients with TNAC vs other TNBC and compare the effect of chemotherapy and radiotherapy in this situation. Comparatively, our study included a large population of only TNAC and TNBC patients, and multivariable Cox regression and competing risk models were used for statistical analysis. Thus, the power of the analysis in our study is convincing.

In addition, the current study found that TNAC patients were associated with an older age, a lower proportion of black race, a lower tumor grade, and a lower T stage than TNBC patients. Most of these findings were consistent with previous studies. Mills et al found that patients with AC more often present in older women with lower grade and T stage,^8^ which is consistent with our findings. According to two retrospective studies, the proportion of lymph node metastasis was significantly lower in patients with AC than in patients with IDC,^11^, ^15^ while our results showed no difference in N stage. In the current study, the results showed that TNAC patients were less likely to receive chemotherapy, while chemotherapy was associated with improved survival in TNAC patients. This result was in accordance with a previously published study.^16^

Inevitably, there were several limitations to the present study. First, this is a retrospective study of the SEER database, so the selection biases might limit the validity of this study. Second, the HER2 status was not included in the SEER data until 2010. Thus, the follow‐up period was limited. Third, it has been widely accepted that AC is defined by a combination of morphologic (apocrine morphology in > 90% of tumor cells) and immunohistochemical (ER‐ and PR‐negative and AR‐positive) characteristics.^17^ However, the AR status was not an essential criterion for the diagnosis of AC; therefore, its expression was not recorded routinely in the SEER database. Additionally, the SEER data do not offer detailed information on chemoradiotherapy regimens, biological targeted therapy, and so on. This information might also affect the survival of breast cancer patients. Finally, some patients did not have clear information about receiving chemotherapy and/or radiation therapy in this study due to the limitations of SEER database coding. We divided this population into patients with treatment and without treatment, so this might reduce the statistical power of the categorical variables. These limitations may have contributed to study bias and undermined the power of the analysis.

## 5 CONCLUSIONS

In the present study, we found that TNAC has unique clinicopathological characteristics. After investigation, our results showed that patients with TNAC have better survival outcomes compared to patients with TNBC. Chemotherapy was associated with improved survival in TNAC. However, radiotherapy was not associated with improved prognosis in TNAC. These findings not only enhance the comprehension of the clinicopathological features and prognostic factors of this rare carcinoma but also provide a basis for the development of therapeutic guidelines for TNAC.

## CONFLICT OF INTEREST

The authors declare that they have no conflict of interest.
